# The effect of dexmedetomidine on intraoperative blood glucose homeostasis: secondary analysis of a randomized controlled trial

**DOI:** 10.1186/s12871-021-01360-3

**Published:** 2021-05-07

**Authors:** Chun-Jing Li, Bo-Jie Wang, Dong-Liang Mu, Dong-Xin Wang

**Affiliations:** Department of Anesthesiology, Peking University First Hospital, Beijing, China

**Keywords:** Dexmedetomidine, Intraoperative hyperglycemia, Elderly, Non-cardiac surgery

## Abstract

**Purpose:**

To investigate the effect of dexmedetomidine on intraoperative blood glucose hemostasis in elderly patients undergoing non-cardiac major surgery.

**Methods:**

This was secondary analysis of a randomized controlled trial. Patients in dexmedetomidine group received a loading dose dexmedetomidine (0.6 μg/kg in 10 min before anaesthesia induction) followed by a continuous infusion (0.5 μg/kg/hr) till 1 h before the end of surgery. Patients in control group received volume-matched normal saline at the same time interval. Primary outcome was the incidence of intraoperative hyperglycemia (blood glucose higher than 10 mmol/L).

**Results:**

303 patients in dexmedetomidine group and 306 patients in control group were analysed. The incidence of intraoperative hyperglycemia showed no statistical significance between dexmedetomidine group and control group (27.4% vs. 22.5%, RR = 1.22, 95%CI 0.92–1.60, *P* = 0.167). Median value of glycemic variation in dexmedetomidine group (2.5, IQR 1.4–3.7, mmol) was slightly lower than that in control group (2.6, IQR 1.5–4.0, mmol), *P* = 0.034. In multivariable logistic analysis, history of diabetes (OR 3.007, 95%CI 1.826–4.950, *P* < 0.001), higher baseline blood glucose (OR 1.353, 95%CI 1.174–1.560, *P* < 0.001) and prolonged surgery time (OR 1.197, 95%CI 1.083–1.324, *P* < 0.001) were independent risk factors of hyperglycaemia.

**Conclusions:**

Dexmedetomidine presented no effect on intraoperative hyperglycemia in elderly patients undergoing major non-cardiac surgery.

**Trial registration:**

Present study was registered at Chinese Clinical Trial Registry on December 1, 2015 (www.chictr.org.cn, registration number ChiCTR-IPR-15007654).

## Introduction

Glucose homeostasis is profoundly disrupted in perioperative settings which is mainly manifested as hyperglycemia and glycemic variability [1]. The incidence of intraoperative hyperglycemia varies from 3% in non-diabetic patients to 15.3% in diabetic patients [2]. It reaches up to 49% in patients who undergoing major non-cardiac surgery [3]. More than 90% of patients suffer glycemic variation with a median magnitude of 5.5 mmol/L during surgery [4]. Both hyperglycemia and magnitude of glycemic variation are related with poor patient’s outcome, such as increased risk of complications (i.e., delirium, infection, acute kidney injury, atrial fibrillation, and 30-day readmission rate) and mortality [3, 5–10].

Surgery related stress response is considered as the key factor of intraoperative dysglycemia [11]. Surgery enhances sympathetic stimulation and subsequently increases levels of the hormones promoting glycogen synthesis, such as catecholamines, cortisol, glucagon, and growth hormones [11, 12]. This escalation leads to an increase in endogenous glucose production via gluconeogenesis and glycogenolysis. Stress response also triggers excessive elevation of circulating proinflammatory cytokines (i.e., interleukins and tumor necrosis factor) [11]. These cytokines result in transient insulin resistance and impairment of insulin signaling pathway which impede glucose metabolism and utility [11, 13].

Dexmedetomidine is a highly selective α-2 adrenergic agonist. Available evidences showed that perioperative application of dexmedetomidine could inhibit stress response and decrease the concentration of miscellaneous stress modulators, i.e., catecholamine and cortisol [14]. In surgical patients (i.e., spine and abdominal surgery), dexmedetomidine could decrease the incidence of hyperglycemia and alleviate glycemic variation [15–17]. However, opposing data indicates that the role of dexmedetomidine in glycemic control is uncertain. In a dose–response analysis, lower dose of dexmedetomidine decreased occurrence of hyperglycemia but higher dosage increased the risk of hyperglycemia in patients undergoing major gastrointestinal surgery [18]. This phenomenon was also observed in pediatric surgical patients [19, 20]. Animal studies showed that dexmedetomidine elevated glucose level via α-2A adrenoceptor which played an important role in regulation of insulin secretion and sympathetic output [21, 22].

Present study was designed to investigate the effect of intraoperative dexmedetomidine on glucose hemostasis in elderly patients undergoing non-cardiac major surgery.

## Materials and methods

This was secondary analysis of a randomized controlled trial which was approved by Clinical Research Ethics Committee of Peking University First Hospital (2015–987) and registered with Chinese Clinical Trial Registry on December 1, 2015 (www.chictr.org.cn, registration number ChiCTR-IPR-15007654) [23]. Written informed consents were obtained from all patients or their legal representatives in original trial. Present study was carried out in accordance with CONSORT 2010 guidelines and Declaration of Helsinki.

### Participants and baseline data collection

Elderly (age ≥ 60 years) patients who underwent selective major non-cardiac surgery with expected duration ≥ 2 h under general anaesthesia were included. Patients who met any of the following criteria were excluded: (1) history of psychiatric disease, i.e., schizophrenia, epilepsy or Parkinson’s disease; (2) visual, hearing, language or other barrier that impeded communication and preoperative delirium assessment; (3) history of traumatic brain injury or neurosurgery; (4) severe bradycardia (heart rate less than 40 beats per minute), sick sinus syndrome, or atrioventricular block of degree 2 or above without pacemaker; (5) severe hepatic dysfunction (Child–Pugh grade C); (6) renal failure (requirement of renal replacement therapy); (7) neurosurgery.

### Randomization and allocation

In this two-armed parallel study, patients were randomized to dexmedetomidine group and control group in a ratio of 1:1. Random numbers were generated by using SAS statistical package version 9.3 (SAS Institute, Cary, NC, USA) with a block size of 4.

### Masking

Opaque envelopes were used to seal random number and kept by a study coordinator who was not involved in patient recruitment, data collection, perioperative care and postoperative follow-up.

Study drugs were prepared by the coordinator according to the randomization results. The study drugs, either 200 μg (2 ml) dexmedetomidine or 2 ml normal saline, were diluted into 50 ml with normal saline (with a final concentration of 4 μg/ml for dexmedetomidine). All study drugs were colourless solution provided in syringes of the same size and brand.

Blinding method of randomization and study drug were masked from patients, investigators who performed data collection and postoperative follow-up, and related healthcare providers. Blinding was maintained throughout the study period.

To ensure patients’ safety, the group allocation could be unmasked in the occurrence of severe adverse events or any unexpected deterioration in the patient’s clinical status. These situations were documented in the case report forms.

### Intervention, anaesthesia and perioperative care

For patients in dexmedetomidine group, a loading dose of dexmedetomidine (0.15 ml/kg, i.e., 0.6 μg/kg) was administered during a 10-min period before anaesthesia induction and then was followed by a continuous infusion at a rate of 0.125 ml/kg/hr (i.e., 0.5 μg/kg/hr) till 1 h before the end of surgery. For patients in control group, volume-matched normal saline was administered at the same rate for the same duration.

To ensure patient’s safety, study drug infusion could be slowed down or stopped by the attending anaesthesiologists in the following conditions: (1) severe bradycardia or hypotension which did not improve after routine treatment; (2) new onset atrioventricular block which did not improve after routine treatment; or (3) other conditions that anaesthesiologists considered necessary. Reasons that led to any protocol deviations were recorded. These patients were included in the intention-to-treat analysis but excluded from the per-protocol analysis.

Anesthesia induction and maintenance were administrated with propofol and sufentanil as well as inhalation of a 1:1 nitrous oxide-oxygen mixture. The aim of anesthesia depth was to maintain Bispectral index (BIS) value between 40 and 60. Non-depolarizing neuromuscular blocking drugs (i.e., rocuronium) were administered for muscle relaxation. Fluid infusion and blood transfusion were performed according to routine practice. Blood pressure was maintained within 20% from baseline and nasopharyngeal temperature between 36.0 and 37.0 °C.

All patients were transferred to the post-anaesthesia care unit (PACU) or the intensive care unit (ICU) before being sent back to general wards. Patient-controlled intravenous analgesia (PCIA) was provided for postoperative analgesia.

### Outcome assessment

#### Primary outcome

Primary outcome was the incidence of intraoperative hyperglycaemia. In consistence with consensuses, hyperglycaemia was defined as serum blood glucose higher than 10 mmol/L at any time during surgery [24, 25].

Blood glucose values were read from arterial blood gas analyser (GEM® Premier 3000, Instrumentation Laboratory, MA, USA). Blood samples were obtained from intra-arterial lines before beginning of surgery and then at 1-h interval till the end of surgery. All patients received at least two arterial blood gas testes during surgery.

#### Secondary outcome

Secondary endpoints included glycemic variation and risk factors of intraoperative hyperglycemia. Glycemic variation was defined as the difference between the highest and lowest perioperative glucose levels during surgery [26].

Baseline and intraoperative variables were analyzed to identify risk factors of hyperglycemia by univariate and multivariable logistic regression analysis.

### Statistical analysis

#### Sample size calculation

Sample size calculation in previous study was based on the hypothesis that intraoperative dexmedetomidine could decrease the incidence of postoperative delirium (309 patients in dexmedetomidine group and 310 patients in control group) [23]. As a secondary analysis, we planned to investigate the effect of dexmedetomidine on intraoperative hyperglycemia. Thus, we excluded patients without record of intraoperative blood glucose record. We finally enrolled 303 patients in dexmedetomidine group and 306 patients in control group.

#### Outcome analysis

The normality of continuous data was tested in prior. Continuous data with normal distribution were compared with the independent sample T-test. Continuous data with non-normal distribution were compared with the independent sample Mann–Whitney U test. Categorical data were compared with the Chi-squared test.

For primary outcome, the incidence of intraoperative hyperglycemia was presented as number (percentage). Estimated effect size was reported in the form of relative risk (RR) with 95% confidence interval (CI) both in intention-to-treat analysis and per protocol analysis. Subgroup analyses were also tested based on age, sex, history of diabetics, site of surgery and type of surgery.

The incidence of glycemic variation was presented in number (percentage) and analysed by Chi-squared test. Univariate analysis was firstly used to analyse the underlying relationship between baseline and intra-operative variables and hyperglycemia. Variables with *P* < 0.1 were entered multivariate analysis to identify independent risk factors of hyperglycemia. Intervention with dexmedetomidine was compulsorily analysed in univariate and multivariable analysis.

Statistical analyses were done with SPSS 14.0 (SPSS, Inc., Chicago, IL) and SAS 9.3 (SAS Institute, Cary, NC, USA). All tests were two tailed and P value less than 0.05 was considered as statistically significant.

## Results

### Participants

**Fig. 1:**
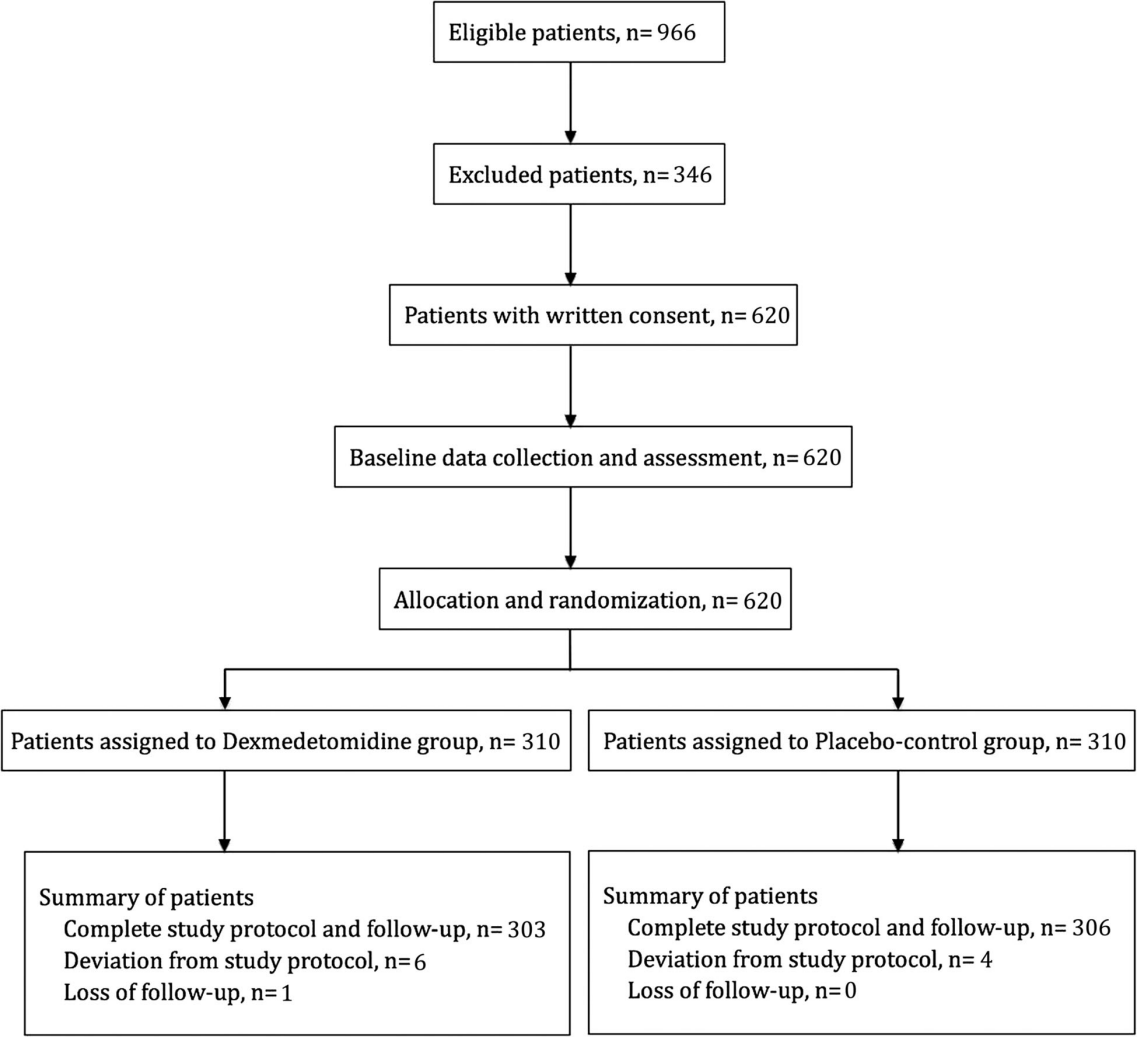
Flowchart of present study

During study period, a total of 620 patients were enrolled and randomized (Fig. 1). In dexmedetomidine group, 1 patient withdrew consent before administration of study drug and 6 patients had no record of intraoperative blood glucose. In the control group, 4 patients had no record of intraoperative blood glucose. Modification of infusion rate of study drug happened in 13 patients in dexmedetomidine group and 8 patients in control group (*P* = 0.257). One patient in the control group died on postoperative day 28.

Baseline variables were comparable between the two groups (Table 1). Patients in dexmedetomidine group consumed less dosage of propofol and sufentanil than in control group (*P* < 0.001 and *P* = 0.012, respectively), whereas anesthesia depth was similar between the two groups (*P* = 0.149), Table 2. Urine output was higher in dexmedetomidine group than in control group (*P* < 0.001), Table 2.

**Table 1:** Baseline data

Variables	Dexmedetomidine group (*n* = 303)	Control group (*n* = 306)
Age, mean (SD), year	69.1 (6.6)	69.0 (6.4)
BMI, Kg/m^2^, mean (SD)	24.1 (3.2)	24.1 (3.4)
BMI ≥ 30, n (%)	12 (4.0)	12 (3.9)
Female, n (%)	179 (59.1)	186 (60.8)
Preoperative comorbidity, n (%)		
Hypertension	143 (47.9)	144 (46.7)
Coronary artery disease	43 (14.2)	49 (16.0)
Arrhythmia	24 (7.9)	30 (9.8)
Congestive heart failure	0 (0.0)	2 (0.7)
Stroke	27 (8.9)	33 (10.8)
Diabetics	70 (23.1)	62 (20.3)
Hyperlipidemia	8 (2.6)	13 (4.2)
COPD	4 (1.3)	5 (1.6)
ASA classification, n (%)		
I	35 (11.6)	41 (13.4)
II	240 (79.2)	228 (74.5)
III	28 (9.2)	37 (12.1)
Baseline blood glucose, mean (SD), mmol/L	6.0 (1.9)	5.9 (1.5)
Baseline blood glucose grade, n (%)		
≤ 6.1 mmol/L	218 (71.9)	219 (71.6)
6.1–7.0 mmol/L	40 (13.2)	39 (12.7)
≥ 7.0 mmol/L	45 (14.9)	48 (15.7)
CCI, median (IQR), score ^a^	4 (4, 5)	4 (4, 5)

*BMI* body mass index, *SD* standard deviation, *COPD* chronic obstructive pulmonary disease, *ASA* American Society of Anesthesiologists, *CCI* Charlson Comorbidity Index, *IQR* interquartile range

^a^ Score ranges from 0–37, with higher score indicating worse prognosis

**Table 2:** Intra- and postoperative data

Variables	Dexmedetomidine group (*n* = 303)	Control group (*n* = 306)
Duration of anesthesia, mean (SD), h	4.8 (1.8)	4.9 (2.0)
Duration of surgery, mean (SD), h	3.6 (1.8)	3.6 (1.8)
Intraoperative drugs		
Study drug, median (IQR), ml	30.0 (23.0, 38.0)	29.0 (23.0, 38.0)
Propofol, median (IQR), mg	817 (600, 1102)	960 (669, 1320)
Sufentanil, median (IQR), μg	72.0 (55.0, 93.0)	78.5 (60.0, 106.0)
Use of tropisetron, n (%)	268 (88.4)	263 (85.9)
Use of NSAIDs, n (%) ^a^	11 (3.6)	10 (3.3)
Use of glucocorticoids, n (%)	298 (98.3)	297 (97.1)
Low-dose glucocorticoids ^b^	295 (97.4)	295 (96.4)
High-dose methylprednisolone ^c^	3 (1.0)	2 (0.7)
Average BIS value, mean (SD) ^d^	50.5 (3.7) (*n *= 291)	51.0 (4.9) (*n* = 294)
Average MAP, mean (SD), mmHg	79.3 (20.2)	79.9 (22.0)
Location of surgery, n (%)		
Intra-thoracic	56 (18.5)	53 (17.3)
Intra-abdominal	200 (66.0)	221 (72.2)
Spinal	47 (15.5)	32 (10.5)
Type of surgery, n (%)		
Thoraco-laparoscopic	230 (75.9)	248 (81.0)
Open thoraco-abdominal/spinal	75 (24.1)	58 (19.0)
Total fluid infusion, median (IQR), ml ^e^	2300 (1700, 3100)	2250 (1600, 3350)
Allogenic red blood cells, n (%)	20 (6.6)	24 (7.8)
Urine output, median (IQR), ml	600 (300, 900)	400 (250, 650)
Estimated blood loss, median (IQR), ml	100 (50, 300)	100 (50, 300)
No. of patients with complications, n (%) ^f^	72 (23.8)	98 (32.0)
Postoperative LOS, median (IQR), day	8 (6, 12)	8 (6, 11)

*SD* standard deviation, *IQR* interquartile range, *NSAIDs* non-steroid anti-inflammatory drugs, *BIS* Bispectral Index, *MAP* mean arterial blood pressure, *No.* number, *LOS* length of stay

^a^ Included parecoxib (40 mg) or flurbiprofen axetil (50 mg), administered before the end of surgery

^b^ Dexamethasone (5–10 mg) or methylprednisolone (40 mg) for the prevention of postoperative nausea and vomiting

^c^ Methylprednisolone 500–1000 mg administered during spinal surgery

^d^ Monitored with Bispectral Index (BIS) with data collected at 1-min interval from end of anesthesia induction to end of surgery

^e^ Included hydroxyethyl starch and/or succinylated gelatin

^f^ Postoperative complications included delirium, ischemic cerebrovascular infarction, acute coronary syndrome, congestive heart failure, new onset atrial fibrillation, deep venous thrombosis, pneumonia, respiratory failure, asthma, acute kidney injury, and surgery-related complications (i.e., gastrointestinal hemorrhage, anastomotic leak and sepsis)

### Primary outcome

**Fig. 2:**
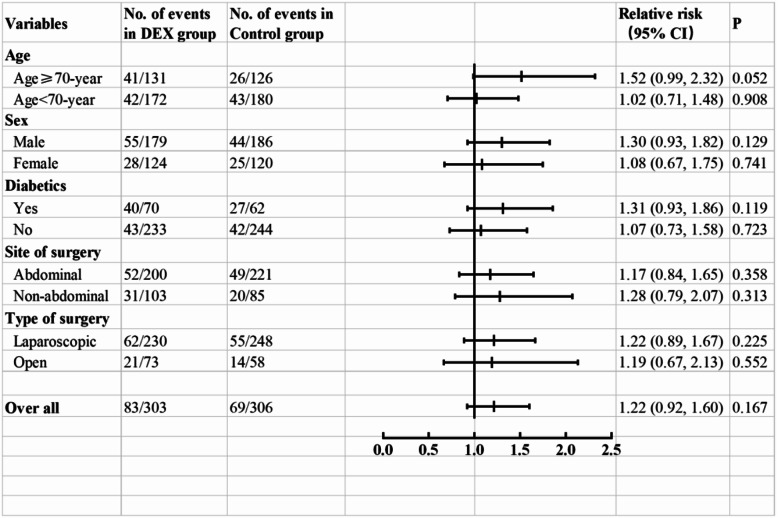
Subgroup analysis of primary outcome. There were no significant interactions between dexmedetomidine and hyperglycemia, even in subgroup analysis of any predefined factors, i.e. sex, age, history of diabetic, site of surgery and type of surgery type (All *P* value > 0.05). DEX = dexmedetomidine; CI = confidence interval

The median of highest blood glucose in dexmedetomidine group was 8.7 (IQR 7.7–10.2) mmol/L whereas 8.4 (IQR 7.1–9.8) mmol/L in control group (*P* = 0.474). The incidence of intraoperative hyperglycaemia was about 27.4% (83/303) in dexmedetomidine group which had no statistical difference in comparison with 22.5% (69/306) of control group (RR 1.22, 95%CI 0.92–1.60, *P* = 0.167), Fig. 2. The result was similar in per protocol analysis, 27.9% (81/290) in dexmedetomidine group vs. 22.5% (67/298) in control group, RR = 1.242, 95%CI 0.939–1.644, *P* = 0.128.

In subgroup analysis, there was no significant relationship between dexmedetomidine and hyperglycaemia on predefined factors, i.e., sex, age, history of diabetic, site of surgery and type of surgery type, Fig. 2.

### Intraoperative glycemic variation

**Fig. 3:**
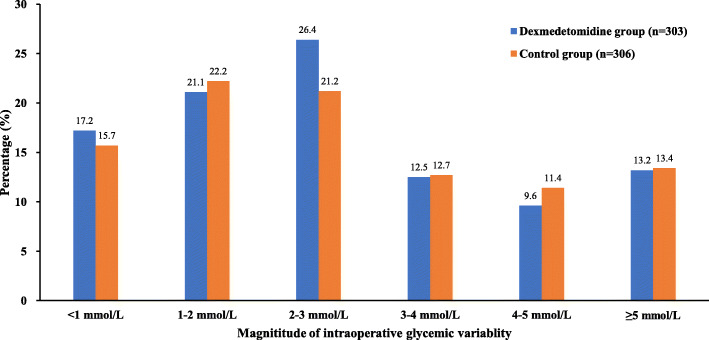
Intraoperative glycemic variability. The magnitude of intraoperative glycemic variability was divided into 6 groups and the frequencies showed no statistical difference between two groups (*P* = 0.581)

The median of glycemic variation in dexmedetomidine group was slightly less than that of control group (2.5 vs. 2.6 mmol/L, *P* = 0.034). The magnitude of glycemic variation was divided into six range groups. The frequencies were presented in Fig. 3 and showed no statistical difference between two groups (*P* = 0.581).

### Risk factors of intraoperative hyperglycaemia

Both in univariate and multivariate analysis, use of dexmedetomidine was not related with hyperglycaemia (OR 1.322, 95%CI 0.881–1.983, *P* = 0.178), Table 3. History of diabetics (OR 3.007, 95%CI 1.826–4.950, *P* < 0.001), higher baseline blood glucose (OR 1.353, 95%CI 1.174–1.560, *P* < 0.001) and prolonged surgery time (OR 1.197, 95%CI 1.083–1.324, *P* < 0.001) were independent risk factors for intraoperative hyperglycemia.

**Table 3:** Risk factors of intraoperative hyperglycemia

Variables	Univariate analysis		Multivariable analysis	
	Odds ratio (95% CI)	*P*	Odds ratio (95% CI)	*P*
History of diabetics (yes)	4.754 (3.142, 7.192)	< 0.001	3.007 (1.826, 4.950)	< 0.001
ASA grade (per grade increase)	1.397 (0.950, 2.054)	0.089	–	–
Baseline blood glucose (per mmol/L increase)	1.579 (1.384, 1.802)	< 0.001	1.353 (1.174, 1.560)	< 0.001
Use of dexmedetomidine (yes)^a^	1.296 (0.897, 1.873)	0.168	1.322 (0.881, 1.983)	0.178
Anesthesia time (per hour increase) ^b^	1.174 (1.070, 1.289)	0.001	–	–
Surgery time (per hour increase) ^b^	1.185 (1.073, 1.308)	0.001	1.197 (1.083, 1.324)	< 0.001
Blood loss (per 50 ml increase)	1.087 (1.023, 1.154)	0.007	–	–

*Kg* kilogram, *ASA* American Society of Anesthesiology, *CI* confidence interval

^a^ Use of dexmedetomidine during surgery was compulsorily analyzed by univariate and multivariate analysis

^b^ There was correlation between anesthesia time and surgery time (Pearson coefficient = 0.969, *P* < 0.001). Only surgery time entered multivariate analysis

## Discussion

Present study found that application of dexmedetomidine had no effect on blood glucose hemostasis in elderly patients undergoing non-cardiac major surgery.

Hyperglycemia has been proposed highly related with poor patient’s outcome, but the definition of intraoperative hyperglycemia is still inconclusive [3, 5–10]. The following criteria has been used in literatures, such as ≥ 8.3 mmol/L, ≥ 10 mmol/L, and ≥ 11.1 mmol/L [5, 24, 25, 27]. The difference in definition significantly influences the homogeneity of results. In present study, we adopted 10 mmol/L as the cut-off point to diagnose intraoperative glycemia in line with guidelines and expert consensus [24, 25].

The effect of dexmedetomidine on intraoperative blood glucose is still uncertain. One meta-analysis showed that infusion of dexmedetomidine could decrease intraoperative blood glucose levels with a mean difference of 1 mmol/L in comparison with control groups, but these results presented significant heterogeneity (*I*^2^ = 97%) [17]. In a pilot study of diabetic patient, intraoperative dexmedetomidine infusion maintained blood glucose levels at a constant level with reference to baseline within 24 h postoperatively and lowered the incidence of hyperglycemia in comparison with control group [15]. We also noticed that the median value of glycemic variation was slightly lower than that of control group (median difference 0.1 mmol/L) in present study, but this seemed to be no clinical relevance.

The association between dexmedetomidine and blood glucose can be influenced by the following factors. First, the effect of dexmedetomidine on blood glucose is dose dependent. In patients undergoing abdominal surgery, patients were divided into three groups and received low, medium and high dosages of dexmedetomidine respectively [18]. In low dosage group, perioperative blood glucose were well regulated in non-diabetic patients whereas higher dosages of dexmedetomidine increased the incidence of hyperglycemia and bradycardia [18]. Evidences in pediatric patients also showed that the elevation of glucose is depended on the dosage of dexmedetomidine [19]. Second, dexmedetomidine could stimulate glucose elevation via α-2A receptor which might overweight its effect of stress alleviation [21, 22]. Third, high dose of dexmedetomidine increased the risk of adverse events (such as hypotension and severe bradycardia) which might induce marked hyperglycemia [28].

In present study, we found that history of diabetics, higher baseline blood glucose and prolonged surgery time were independent risk factors of intraoperative risk factors. This result was also supported by other studies [29].

Strength of present study was a relatively large sample size than previous studies [17]. We also conducted subgroup analysis to analyze the relationship between dexmedetomidine and blood glucose in different populations.

One limitation was that we excluded patients who were not suitable to receive dexmedetomidine, such as severe arrythmia and hepatic dysfunction. This excluded patients with severe disease and limited the generality of our result. Another limitation was that postoperative blood glucose was not analyzed.

## Conclusions

For elderly patients undergoing major non-cardiac surgery, intraoperative administration of dexmedetomidine had no effect on the incidence of hyperglycemia. The effect of dexmedetomidine on hyperglycemia deserves further study. For example, if the dosage and administration time of dexmedetomidine will influence the incidence of intra- and post-operative hyperglycemia.

## Data Availability

The datasets used and analysed during the current study are available from the corresponding author (DL Mu, mudongliang@bjmu.edu.cn) on reasonable request.

## Abbreviations

BIS: Bispectral index
PACU: Post-anaesthesia care unit
ICU: Intensive care unit
PCIA: Patient-controlled intravenous analgesia
RR: Relative risk
IQR: Interquartile range
OR: Odds ratio
